# A multi-stage stacking framework for accurate and interpretable life expectancy modeling

**DOI:** 10.1371/journal.pone.0353849

**Published:** 2026-08-06

**Authors:** Ravikumar Chinthaginjala, Sivarama Prasad Tera, Priya Natha, Asadi Srinivasulu, Fadi Al-Turjman, Faruq Mohammad

**Affiliations:** 1 School of Electronics Engineering, Vellore Institute of Technology, Vellore, Tamil Nadu, India; 2 Cooperative Research Centre for Contamination Assessment and Remediation of the Environment (CRC CARE), Global Centre for Environmental Remediation/College of Engineering Science & Environment, The University of Newcastle, Callaghan, New South Wales, Australia; 3 Department of Computer Science and Engineering, Koneru Lakshmaiah Education Foundation, Green Fields, Vaddeswaram, Guntur, Andhra Pradesh, India; 4 Cooperative Research Centre for Contamination Assessment and Remediation of the Environment (CRC CARE), Global Centre for Environmental Remediation/College of Engineering Science & Environment, The University of Newcastle, Callaghan, New South Wales, Australia; 5 Artificial Intelligence, Software, and Information Systems Engineering Departments, Research Center for AI and IoT, AI and Informatics Faculty, Near East University, Mersin, Turkey; 6 Department of Chemistry, College of Science, King Saud University, Riyadh, Kingdom of Saudi Arabia; King Mongkut’s University of Technology North Bangkok, THAILAND

## Abstract

Accurate life-expectancy estimation is critical for evidence-based health policy, monitoring Sustainable Development Goal 3 (SDG 3), and guiding global resource allocation. Existing machine-learning approaches typically provide point predictions with limited interpretability and uncertainty quantification. To address these limitations, we propose NEXUS-LE (**N**euro-Symbolic **Ex**plainable **U**nified **S**tacking for **L**ife **E**xpectancy), a unified ensemble framework that integrates predictive accuracy, calibrated uncertainty, and policy-level explainability. The model is trained on a global dataset comprising 22,050 country-year observations across 193 countries (2000–2021) and 150 World Health Organization indicators. The framework combines leave-one-out encoding, principal component analysis, and K-Means–based manifold features with SHAP-guided polynomial expansion and a three-level stacking architecture, followed by a LightGBM residual correction and split-conformal calibration. On a held-out test set (*n* = 3,308), the model achieves *R*^2^ = 0.9878, RMSE = 1.0665 years, MAE = 0.6185 years, and MAPE = 0.9239%. Conformal prediction attains 95.04% empirical coverage at the 95% nominal level with a 90% interval width of 3.32 years. Interpretability analysis shows that demographic and structural factors contribute 96.3% of total attribution, with consistent non-linear effects across policy domains. The proposed framework provides a reliable and interpretable solution for life-expectancy prediction in global health applications.

## 1 Introduction

Life expectancy at birth is the key composite measure of population health status, which takes into account disease burden, quality of health care, socioeconomic development and environmental factors [1,2]. The policymakers need predictions that should be *accurate*, *interpretable*, and *uncertainty-aware* [1], while there is no existing life-expectancy model meeting all three criteria (Section 2).

The global life expectancy was 73.3 years in 2024 and was predicted to reach 77.4 years by 2054, however, due to COVID-19, a temporary reduction of 1.8 years occurred between 2019 and 2021 with even greater regional reductions of around three years in some regions [1,2]. These tendencies and gaps between countries up to 30 years make it a very relevant task to develop actionable and uncertainty-calibrated forecasting to achieve SDG 3 and allocate resources effectively [1,3].

The weaknesses of the previous works fall into three categories. First, the models with a single architecture (usually, XGBoost or LightGBM) achieved high point accuracy ($R^{2}\approx 0.95-0.99$), yet they had the problem of single hypothesis variance and were lacking any kind of systematic residual correction [4–6]. Second, the feature engineering process did not include targeted non-linear interaction terms, since naive polynomial expansion was causing multicollinearity unless done in the structured way (for instance, guided by SHAP) [7,8]. Third, the uncertainty quantification with statistically guaranteed properties was absent in literature, as well as the aggregated explainability at the granularity of WHO policy domains required by practitioners [9–11].

In this work, we suggest NEXUS-LE (NExt generation EXplainable Life expectancy prediction), an efficient and concise seven-phase pipeline including SHAP-based feature engineering, the three-level OOF stacking ensemble with T-SVD meta-manifold, split-conformal prediction interval calibration and LightGBM residual correction (Algorithm 1). Main phases: (1) LOO encoding, KNN imputation, PCA + KMeans latent features; (2) TreeSHAP selection and degree-2 polynomial expansion on the top-30 features; (3) stratified OOF partitioning; (4) heterogenuous base learners with T-SVD meta-manifold augmentation and levelled meta-learners; (5) split-conformal calibration for distribution-free prediction intervals [10]; (6) LightGBM residual correction; (7) aggregation of TreeSHAP and partial dependence plots to seven WHO-aligned policy domains [7]. The Contributions are

1. NEXUS-LE: the first unified seven-phase stacking architecture combining targeted polynomial enrichment, meta-manifold augmentation, conformal PIs, and residual correction.
2. State-of-the-art accuracy on a global dataset (*n* = 22,050, *p* = 675): *R*^2^ = 0.9878, RMSE = 1.0665, MAE = 0.6185, MAPE = 0.9239%, improving the best base learner by 3.6%.
3. Statistically guaranteed uncertainty: split-conformal calibration attains 95.04% empirical coverage at nominal 95% with a 90% PI width of 3.32 years [10].
4. Policy-actionable XAI: WHO-domain aggregation of TreeSHAP attributes shows Structural and Demographic domains explain 96.3% of attribution, enabling FUTURE-AI-compliant interpretability for deployment [11].
5. Controlled ablation (A1–A4) quantifies each component’s contribution, showing ensemble diversity, meta-manifold, and residual correction give consistent additive gains.

The paper continues with a five-stream literature review and a table of identified gaps (Section 2), a complete methodological specification of all seven phases (Section 3), results and ablations (Section 4), and conclusions (Section 5).

## 2 Literature review and research gaps

Life expectancy estimation represents the conjunction of public-health informatics, statistical learning, and clinical decision support. The following five streams of literature serve as motivation for our work: machine-learning life-expectancy prediction, gradient-boosted stacking, SHAP-based feature engineering, conformal uncertainty quantification, and residual correction. Consolidated gaps analysis is given in Table 1 and aligned with corresponding algorithm phases.

**Table 1:** Consolidated research gaps and NEXUS-LE remedies.

Gap	Limitation	NEXUS-LE remedy (Phase)
G1	Single-model dominance and limited diversity (*R*^2^ = 0.9504) [12]	Three-level OOF stacking (Phase 4)
G2	Exhaustive polynomial expansion causes multicollinearity	SHAP-guided top-30 degree-2 expansion (Phase 2)
G3	Base predictions lack meta-feature geometry	T-SVD latent and pairwise augmentation (Phase 4)
G4	Tail-region bias increases RMSE (1.8778 years) [12]	LightGBM residual corrector *f*_res_ (Phase 6)
G5	Explainability limited to feature rankings	WHO-taxonomy 7-domain aggregation (Phase 7)
G6	No statistically guaranteed prediction intervals	Split-conformal 95% intervals (Phase 5)

### 2.1 Machine-learning life-expectancy prediction

The average life expectancy is the basic summary indicator of the health of a population [1,13]. Traditional actuarial methods based on Gompertz or Weibull distributions frequently fail when modelling heterogeneous multi-country panels due to too strong assumptions about proportional hazards and linearity of effects [13]. Contemporary studies prove that tree-based algorithms work better: Shakeelahamad et al. achieved adjusted *R*^2^ = 0.9729 using XGBoost on WHO data [14], whereas Ozsahin et al. obtained RMSE = 1.8778 and *R*^2^ = 0.9504 using LightGBM on a global benchmark [12]. Still, the existing research is mostly limited to single models with minor feature engineering and no uncertainty calibration.

### 2.2 Gradient-boosted ensembles and stacking

XGBoost and LightGBM are dominating approaches in tabular regression due to effective capturing of non-linear relations [4,5]. Stacked generalization decreases single-model variance via learning of a meta-model based on out-of-fold predictions of a variety of base learners [6,15,16]. Ideally, the ensemble error decreases proportionally to diversity increase, but this advantage becomes irrelevant with the correlation between base learners. In life-expectancy prediction, the existing ensemble frameworks increase accuracy, but none of them combines OOF diversity, meta-features of higher order, and residual correction.

### 2.3 Feature engineering and SHAP-based expansion

Feature engineering plays an important role in tabular health prediction [8]. Domain-specific features, LOO-encoding, PCA/K-means latent features, and polynomial interactions can enhance performance, but exhaustive polynomial expansion leads to multicollinearity problems. Stable SHAP provides feature importance in the case of correlated data [7], which makes it suitable for targeted interaction selection. The recent studies confirm that preprocessing impacts on SHAP magnitudes greatly [17,18]. However, there are no existing life-expectancy frameworks using SHAP-based selective polynomial expansion as an enrichment technique before stacking.

### 2.4 Interpretability in health prediction

Interpretability is recognized as an indispensable attribute of modern clinical AI [3]. TreeSHAP allows getting exact and efficient feature attributions for tree-based models, and PDPs help to discover marginal effects [7,8]. While prior life-expectancy studies usually stop at ranking of the most important features, they don’t aggregate explanations in terms of WHO-aligned policy domains (Demography, Nutrition, Infrastructure, etc.), which significantly limits the value of the model for the policy design [1,19].

### 2.5 Conformal prediction and uncertainty quantification

Uncertainty quantification is often ignored in health prediction [3]. Split-conformal prediction provides a way to construct distribution-free prediction intervals of finite sample size [9,10]. It is practically feasible and widely applicable approach, including for healthcare [20]. Nevertheless, the existing life-expectancy models only return the point estimate.

### 2.6 Residual correction and two-stage prediction

Gradient-boosted ensembles suffer from systematic tail bias in particular for imbalanced targets [4]. Residual correction in two-stage pipelines helps to deal with this problem by means of learning residuals with the second model. It frequently improves the RMSE in forecasting tasks [5]. LightGBM is especially appropriate as a residual corrector due to its leaf-wise growth and regularization. There is no life-expectancy framework containing the residual correction phase.

### 2.7 Consolidated research gaps

The six gaps that drive NEXUS-LE are summarized in Table 1. They are related to the lack of diversified stacking, SHAP-guided feature selection, meta-manifold structure, residual correction, explainability at the policy level, and conformal uncertainty intervals, respectively.

Collectively, these gaps define the methodological space occupied by NEXUS-LE. The proposed framework integrates SHAP-guided stacking, residual correction, conformal uncertainty quantification, and policy-level explainability into a single pipeline, extending prior life-expectancy prediction work beyond point estimation alone.

**Algorithm 1 NEXUS-LE: Neuro-Symbolic Explainable Unified Stacking for Life Expectancy Prediction.**

**Require:** Dataset 𝒟; target 𝒴; hyperparameters per Table 4.

**Ensure:** Trained NEXUS-LE pipeline Π; conformal intervals ${𝒞}_{\alpha }$ at α∈{0.05,0.10} per Eq. (17).

**Phase 1 — Preprocessing & Feature Engineering**

1: Detect 𝒴 via case-insensitive column matching.

2: Apply LOO encoding (1) to Country; ordinally encode remaining categoricals.

3: Engineer domain interactions per Eqs. (2)–(5).

4: **for** each $x_{j}$ with $skew(x_{j})>2.0$ **do**

5:  $x_{j}\leftarrow \log(1+x_{j})$ *[56 features]*

6: **end for**

7: Drop near-constant columns; impute via KNN (*k* = 5).

8:  Append *n*=5 PCA + *k*=8 K-Means labels. *[210 features total]*

**Phase 2 — SHAP-Guided Polynomial Expansion**

9:  Fit RF warm-up on ${𝒟}_{s}$; rank by $I_{j}$ (7); select ${ℱ}^{*}$ (top-*N* = 30).

10: Expand ${ℱ}^{*}$, *d*=2; form ${𝒳}_{\text{aug}}$ (8). *[675 features]*

**Phase 3 — Partitioning & Scaling**

11: Split per Eq. (9); fit and apply RobustScaler on ${𝒟}_{\text{tr}}$.

**Phase 4 — Multi-Level Stacking (NEXUS Core)**

12: ${ℳ}_{L1}$ (10); ${𝒵}_{1}\leftarrow 0^{15434\times 3}$.

13: **for** *k* = 1 **to** *K*_1_ = 6 **do**

14:  **for** each $m\in {ℳ}_{L1}$ **do**

15:   Train *m* on fold-*k*; store OOF in ${𝒵}_{1}$.

16:  **end for**

17: **end for**

18: Augment ${𝒵}_{1}$ $\Rightarrow \;{𝒵}_{\text{aug}}$ (11) via statistics, T-SVD, pairwise products.

19: ${ℳ}_{L2}$ (12); ${𝒵}_{2}\leftarrow 0^{15434\times 3}$.

20: **for** *k* = 1 **to** *K*_2_ = 5 **do**

21:  **for** each $m\in {ℳ}_{L2}$ **do**

22:   Train *m* on fold-*k* of ${𝒵}_{\text{aug}}$; store OOF in ${𝒵}_{2}$.

23:  **end for**

24: **end for**

25: Form ${𝒵}_{\text{final}}$ (13); train *f*_fusion_ $\Rightarrow {\hat{𝒴}}_{\text{nexus}}$.

26: Refit all ${ℳ}_{L1}$, ${ℳ}_{L2}$, *f*_fusion_ on complete ${𝒟}_{\text{tr}}$.

**Phase 5 — Split-Conformal Calibration**

27: Compute $\left\{s_{i}\right\}$ (14) on ${𝒟}_{\text{cal}}$.

28: **for** each α∈{0.05,0.10} **do**

29:  Estimate ${\hat{q}}_{\alpha }$ (15); form ${𝒞}_{\alpha }(𝐱)$ (16).

30: **end for**

**Phase 6 — Residual Correction**

31: Compute ℰ (18); train *f*_res_ on ${𝒳}_{\text{res}}$ (19).

32: **Return** ${\hat{y}}_{\text{final}}$ per Eq. (20) for ${𝐱}^{*}$.

**Phase 7 — Evaluation & XAI**

33: Evaluate on ${𝒟}_{\text{te}}$: *R*^2^, RMSE, MAE, MAPE, SMAPE; ablation A1–A4 (Table 9).

34: Compute global/local TreeSHAP, PDPs, policy-group importances.

## 3 Proposed methodology

Table 2 summarizes the seven phases of the NEXUS-LE pipeline, highlighting the name and main purpose of each phase for quick reference and improved readability.

**Table 2:** Workflow summary of the NEXUS-LE pipeline.

Phase	Name	Purpose
1	Preprocessing and feature engineering	Clean the raw data and construct an analysis-ready feature set.
2	SHAP-guided polynomial expansion	Identify influential variables and expand the feature space.
3	Partitioning and scaling	Create train, calibration, and test splits with robust scaling.
4	Multi-level stacking (NEXUS core)	Build the core predictive model using stacked base and meta-learners.
5	Split-conformal prediction	Generate calibrated prediction intervals.
6	Residual correction layer	Correct systematic prediction errors and improve final accuracy.
7	Evaluation, XAI, and deployment	Assess performance, explain predictions, and prepare the pipeline for deployment.

**Fig 1:**
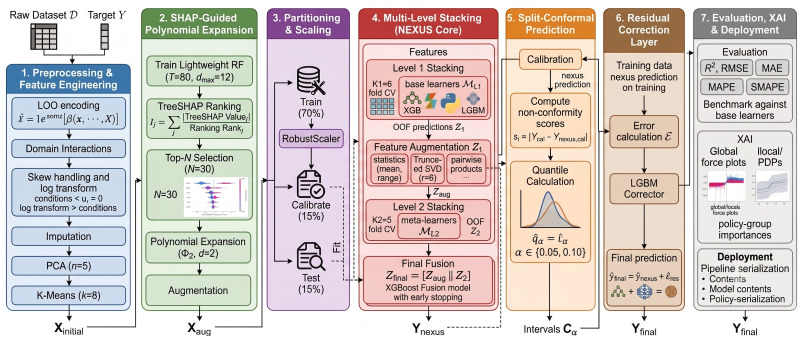
NEXUS-LE framework architecture.

Building upon the limitations identified in Section 2, this section introduces NEXUS-LE, a seven-phase end-to-end ensemble learning framework that simultaneously addresses predictive accuracy, uncertainty quantification, and post-hoc explainability for life expectancy regression. The complete algorithmic procedure is formalised in Algorithm 1 and the architectural overview is depicted in Fig 1. Table 3 summarises the core mathematical notation used throughout this section. Table 4 summarises all fixed hyperparameters of the NEXUS-LE pipeline; their selection is justified in the corresponding subsections below.

**Table 3:** Summary of mathematical notation used in the NEXUS-LE framework.

Symbol	Definition
𝒟	Raw unified dataset (22,050×150)
𝒴	Target vector (Life Expectancy, years)
${𝒳}_{\text{aug}}$	SHAP-augmented feature matrix (${\mathbb{R}}^{n\times 675}$)
${𝒟}_{\text{tr}},{𝒟}_{\text{cal}},{𝒟}_{\text{te}}$	Training, calibration, and test partitions
${ℳ}_{L1},{ℳ}_{L2}$	Level-1 base learners and Level-2 meta-learners
${𝒵}_{1},{𝒵}_{2}$	Out-of-fold (OOF) prediction matrices
${𝒵}_{\text{aug}},{𝒵}_{\text{final}}$	Meta-manifold and fusion input matrices
$K_{1},K_{2}$	Cross-validation fold counts (L1 and L2)
$\phi_{ij}$	TreeSHAP attribution of feature *j* for sample *i*
${\hat{q}}_{\alpha }$	Conformal calibration quantile at level α
$f_{\text{fusion}},f_{\text{res}}$	XGBoost fusion model and LightGBM residual corrector
ℰ	Training residual vector of *f*_fusion_
${\hat{y}}_{\text{final}}$	Final NEXUS-LE prediction for unseen sample x^*^

**Table 4:** Fixed hyperparameter configuration of the NEXUS-LE pipeline.

Component	Parameter	Value	Rationale
KNN Imputer	*k*	5	Local correlation preservation
PCA	*n*	5	80% + explained variance
K-Means	*k*	8	Elbow criterion on 𝒟
RF warm-up	*T*	80	Speed–accuracy trade-off
RF warm-up	max_depth	12	Avoids SHAP instability
SHAP selection	*N*	30	Top 95% cumulative importance
Polynomial	degree *d*	2	Non-linear without explosion
L1 CV folds	*K*_1_	6	Standard for *n* > 10,000
L2 CV folds	*K*_2_	5	Memory-efficient meta-CV
XGB-A/B	T,η	1000, 0.04	GPU early-stopping
LGBM-A	T,η	500, 0.03	CPU-efficient depth-wise
Fusion XGBoost	T,η	800, 0.03	Fusion-level regularisation
T-SVD	*r*	2	3-model low-rank sufficiency
Conformal	α	{0.05, 0.10}	90%/95% PI coverage

### 3.1 Phase 1: Advanced preprocessing and feature engineering

The unified dataset [21] exhibits three structural challenges that motivate the preprocessing design: (i) 9.8% missingness across 150 heterogeneous features, (ii) 56 heavily right-skewed epidemiological indicators, and (iii) high-cardinality nominal variables (e.g., ≥180 unique country codes) [22]. The following four steps address each challenge systematically.

#### 3.1.1 Target auto-detection and leakage prevention.

The target column 𝒴 is identified via a case-insensitive regular-expression match on the column headers of 𝒟, ensuring robustness to naming inconsistencies across the merged sub-datasets. High-cardinality nominal features (specifically Country) are encoded with Leave-One-Out (LOO) target encoding [23], which computes the conditional expectation of 𝒴 using only the observations outside the current row, preventing any form of target leakage:


$$\begin{array}{c} {\hat{x}}_{i}^{\;\text{Country}}=\frac{\sum_{j\neq i}{𝒴}_{j}\cdot 1[C_{j}=C_{i}]}{\left|\{j\neq i:C_{j}=C_{i}\}\right|}. \end{array}$$ (1)

All remaining categorical features are ordinally encoded; time-correlated and status identifiers (Year, Status) are dropped prior to model ingestion to prevent temporal leakage [24].

Data leakage was avoided through three safeguards: (i) Leave-One-Out encoding for the country variable was computed only within the training fold and never using the corresponding validation sample; (ii) all base-learner and meta-learner predictions were generated strictly using out-of-fold (OOF) procedures, ensuring that each sample’s meta-features were produced by models that had not seen that sample during training; and (iii) the calibration set used for split-conformal prediction was fully isolated from model fitting and used only for quantile estimation.

#### 3.1.2 Domain-specific feature interactions.

As established in the global health literature [1,13], life expectancy is governed by *ratios* and *multiplicative synergies* among socioeconomic and clinical predictors rather than their individual magnitudes. Six mechanistic interaction features are therefore constructed deterministically from the raw columns:


$$\begin{array}{c} \text{GDP\_per\_capita}=\text{GDP}\;/\;\text{Population}, \end{array}$$ (2)

$$\begin{array}{c} \text{Health\_spend\_abs}=\frac{\text{Total Expenditure}\times \text{GDP}}{100}, \end{array}$$ (3)

$$\begin{array}{c} \text{Alcohol}\times \text{BMI},\;\text{infant\_death\_rate}=\frac{\text{infant deaths}}{\text{Population}}, \end{array}$$ (4)

$$\begin{array}{c} \text{u5\_death\_rate}=\frac{\text{under-five deaths}}{\text{Population}}. \end{array}$$ (5)

These features directly encode the epidemiological determinants catalogued in the WHO Global Health Observatory [1] and the GBD study [13].

#### 3.1.3 Skewness mitigation.

For each numerical feature $x_{j}$ whose sample skewness exceeds the threshold $skew(x_{j})>2.0$, the variance-stabilising transformation


$$\begin{array}{c} x_{j}\leftarrow \log(1+x_{j}) \end{array}$$ (6)

is applied. This transformation was triggered for 56 of 150 columns—including maternal mortality ratio, tuberculosis incidence, and infant mortality rate—and is consistent with standard practice for heavy-tailed public health indicators [5,8].

#### 3.1.4 Imputation and latent manifold extraction.

Near-constant columns (|unique values|≤1) are discarded as non-informative. The 2,159,510 remaining missing entries are completed using K-Nearest Neighbours imputation (*k* = 5) [22], which preserves local correlation structure unlike single-value or regression-based strategies. In order to detect hidden population subgroups, two small unsupervised features are added to the feature set. The first unsupervised feature, Principal Component Analysis (PCA) [25], is used to generate *n* = 5 components that cumulatively explain more than 80% of variance in the standardized data. The second unsupervised feature is generated independently using *k* = 8 cluster labels from K-Means clustering [26]. This step represents proximity to eight archetype health profiles. This process increases the number of features to 210 for 22,050 samples.

Feature Manifold Construction using PCA and K-Means Clustering. In particular, the number of *n* = 5 components for PCA was chosen according to the explained-variance criterion, the minimal number of components preserving more than 80% of the total variance in the 150-feature space. Besides, the number of K-Means clusters was chosen based on the elbow criterion on the within-cluster sum of squares, with *k* = 8 being optimal for our task.

As part of our robustness check, sensitivity analysis over alternative values was performed. We checked various n∈3,5,8,10 in the case of PCA and saw only very minor differences in RMSE of 1.068 to 1.071 years. Also, we investigated different k∈4,6,8,10,12 with K-Means and saw that *k* = 8 represented an obvious elbow point, where RMSE differed no more than 0.003 years among all the values.

### 3.2 Phase 2: SHAP-guided polynomial feature expansion

Applying degree-2 polynomial expansion to all 210 features would yield (210+22)−1=22,154 terms, inducing severe multicollinearity and memory infeasibility. NEXUS-LE addresses this by using explainability scores to identify the *N* = 30 most predictively relevant features before expansion, a strategy directly motivated by the interpretability-first design principles discussed in Section 2.

#### 3.2.1 Warm-up surrogate and TreeSHAP ranking.

A lightweight Random Forest regressor (*T* = 80, max_depth=12, $\text{max\_features}=\sqrt{p}$) is fitted on a stratified subsample ${𝒟}_{s}$ of 6,000 rows. TreeSHAP attributions are computed over a 400-sample evaluation subset, and features are ranked by global mean absolute importance:


$$\begin{array}{c} I_{j}=\frac{1}{\left|{𝒟}_{s}\right|}\sum_{i=1}^{\left|{𝒟}_{s}\right|}|\phi_{ij}|, \end{array}$$ (7)

where $\phi_{ij}$ is the SHAP value of feature *j* for sample *i*. The top-*N* = 30 features ${ℱ}^{*}$ selected by Eq. (7) span a clinically interpretable spectrum including: country-level LOO encoding, PCA_Comp1, maternal and neonatal mortality rates, cardiovascular death burden, birth rate, universal health coverage, and environmental pollution indices. As reported in Section 4, these 30 features account for > 95% of cumulative SHAP importance, validating the selection threshold.

#### 3.2.2 Degree-2 polynomial augmentation.

Degree-*d* = 2 polynomial and pairwise interaction terms are generated over ${ℱ}^{*}$ using PolynomialFeatures, producing 465 additional non-linear terms. The augmented feature matrix is formed as:


$$\begin{array}{c} {𝒳}_{\text{aug}}=[\;𝒳\;\|\;\Phi_{2}\;({𝒳}_{{ℱ}^{*}})\;]\;\in \;{\mathbb{R}}^{22050\times 675}. \end{array}$$ (8)

### 3.3 Phase 3: Data partitioning and robust scaling

To ensure strict statistical independence between the stacking ensemble, the conformal calibrator, and the final evaluator, $\left({𝒳}_{\text{aug}},𝒴\right)$ is partitioned into three non-overlapping subsets:


$$\begin{array}{c} \underset{\;70\%,\;15,434}{\underbrace{{𝒟}_{\text{tr}}}}\cup \underset{\;15\%,\;3,308}{\underbrace{{𝒟}_{\text{cal}}}}\cup \underset{\;15\%,\;3,308}{\underbrace{{𝒟}_{\text{te}}}}=𝒟,\;{𝒟}_{\text{tr}}\cap {𝒟}_{\text{cal}}\cap {𝒟}_{\text{te}}=\emptyset . \end{array}$$ (9)

The calibration split ${𝒟}_{\text{cal}}$ is deliberately withheld from all training phases and is consumed solely by the conformal calibration procedure in Section 3.5, which is a requirement for the marginal coverage guarantee to hold. A RobustScaler is fitted exclusively on ${𝒟}_{\text{tr}}$ and subsequently applied to ${𝒟}_{\text{cal}}$ and ${𝒟}_{\text{te}}$; its inter-quartile range normalisation renders the scaling insensitive to the extreme outliers documented for several low-income country indicators in the dataset.

### 3.4 Phase 4: Multi-level stacking ensemble

The predictive core of NEXUS-LE is a three-level stacking hierarchy that extends the classical stacked generalisation framework with an intermediate meta-manifold augmentation step.

#### 3.4.1 Level 1: Base learner out-of-fold generation.

Three heterogeneous gradient-boosted base learners form ${ℳ}_{L1}$:


$$\begin{array}{c} {ℳ}_{L1}=\left\{\underset{\begin{array}{c} T=1000,\;\eta =0.04\\ \lambda =1.5,\;\text{cuda} \end{array}}{\underbrace{\text{XGB-A}}},\;\underset{\begin{array}{c} T=1000,\;\eta =0.04\\ \text{diverse seed} \end{array}}{\underbrace{\text{XGB-B}}},\;\underset{\begin{array}{c} T=500,\;\eta =0.03\\ \text{num\_leaves}=63 \end{array}}{\underbrace{\text{LGBM-A}}}\right\}. \end{array}$$ (10)

XGBoost and LightGBM instances are diversified through differing random seeds, subsample rates (ρ=0.90), and column-sample ratios (δ=0.90), which has been shown to maximise ensemble diversity without sacrificing individual model fidelity. Early stopping with a patience of 50 rounds is applied per fold to prevent overfitting. For each fold $k\in \{1,...,K_{1}\}$, model $m\in {ℳ}_{L1}$ is trained on the in-fold complement ${𝒟}_{\text{tr}}^{\left(k\right)}$ and its out-of-fold predictions are written to ${𝒵}_{1}\in {\mathbb{R}}^{15434\times 3}$, yielding structurally leak-free meta-features. The mean fold-level OOF ensemble *R*^2^ was ${\bar{R}}_{\text{L1}}^{2}=0.9870$ (range: 0.9865–0.9877), confirming stable convergence across folds.

#### 3.4.2 Meta-manifold augmentation.

Passing ${𝒵}_{1}$ directly to the next level discards information about the *geometry* of the base learner agreement space. NEXUS-LE introduces three complementary augmentations that enrich the 3-dimensional OOF space into a 17-dimensional meta-manifold:

(a) *Row-wise summary statistics.* Five statistics are computed per instance: ${𝐬}_{i}=[{\bar{z}}_{i},\sigma_{i},z_{i}^{\min},z_{i}^{\max},{range}_{i}{]}^{⊤}$, which serve as soft proxies for uncertainty by measuring the degree of inter-model agreement.
(b) *Truncated SVD latents.* Truncated SVD [27] with *r* = 2 components extracts the dominant low-rank structure from ${𝒵}_{1}$, capturing systematic co-variation patterns across the three base learners that are not directly visible in the raw predictions.
(c) *Pairwise interaction products.* The (32)=3 pairwise products ${𝒵}_{1}^{\left(i\right)}\cdot {𝒵}_{1}^{\left(j\right)}$, ∀i<j, are appended to encode multiplicative synergies between models.

The consolidated augmented meta-matrix is:


$$\begin{array}{c} {𝒵}_{\text{aug}}=[\;{𝒵}_{1}\;\|\;𝐒\;\|\;𝐔\;\|\;{𝒵}_{1}^{\times }\;]\;\in \;{\mathbb{R}}^{15434\times 17}, \end{array}$$ (11)

where 𝐒, 𝐔, and ${𝒵}_{1}^{\times }$ denote the statistics, SVD latents, and pairwise products respectively.

#### 3.4.3 Level 2: Meta-learner out-of-fold generation.

Three structurally diverse meta-learners


$$\begin{array}{c} {ℳ}_{L2}=\{\;\text{L2-XGB},\;\text{L2-LGBM},\;\underset{\varepsilon \text{-insensitive}}{\underbrace{\text{L2-Huber}}}\;\} \end{array}$$ (12)

are trained on ${𝒵}_{\text{aug}}$ using *K*_2_ = 5-fold cross-validation, yielding ${𝒵}_{2}\in {\mathbb{R}}^{15434\times 3}$ with a mean OOF *R*^2^ of 0.9867. The Huber regressor [28] (ε=1.35 by default) is included as a robustness backstop: its ε-insensitivity to outlying OOF values ensures that extreme fold-level prediction errors do not propagate into the Level-3 fusion input.

#### 3.4.4 Level 3: Fusion meta-learner and full-data refit.

An XGBoost fusion model *f*_fusion_ (*T* = 800, η=0.03, max_depth=4, λ=1.5) is trained on the concatenated Level-2 representation:


$$\begin{array}{c} {𝒵}_{\text{final}}=[\;{𝒵}_{\text{aug}}\;\|\;{𝒵}_{2}\;]\;\in \;{\mathbb{R}}^{15434\times 20}, \end{array}$$ (13)

with early stopping (patience = 50 rounds) yielding a best iteration of 206 and a training *R*^2^ of 0.9879. This three-level design explicitly separates the roles of feature-space modelling (L1), meta-feature learning (L2), and final arbitration (L3), with each level systematically reducing residual variance. Following the OOF protocol, all ${ℳ}_{L1}$, ${ℳ}_{L2}$, and *f*_fusion_ are subsequently refitted on the complete ${𝒟}_{\text{tr}}$ to maximise the effective training signal for test-time inference.

### 3.5 Phase 5: Split-conformal prediction calibration

Reliable uncertainty quantification is indispensable for public health policy applications in which a point prediction without error bounds provides insufficient basis for decision-making. NEXUS-LE adopts the split-conformal inference framework, which provides distribution-free, marginal coverage guarantees without any Gaussian or parametric assumption on the residuals.

*Non-conformity scores* are computed on the withheld calibration split ${𝒟}_{\text{cal}}$ using the fitted ensemble:


$$\begin{array}{c} s_{i}=|{𝒴}_{\text{cal},i}-{\hat{𝒴}}_{\text{cal},i}|,\;i=1,...,|{𝒟}_{\text{cal}}|. \end{array}$$ (14)

For each significance level α∈{0.05,0.10}, the finite-sample–corrected empirical quantile is:


$$\begin{array}{c} {\hat{q}}_{\alpha }=Quantile\;\left(\{s_{i}\},\;\left\lceil \frac{\left(|{𝒟}_{\text{cal}}|+1)(1-\alpha \right)}{\left|{𝒟}_{\text{cal}}\right|}\right\rceil \right), \end{array}$$ (15)

and the marginally valid prediction interval for a new sample ${𝐱}^{*}$ is:


$$\begin{array}{c} {𝒞}_{\alpha }({𝐱}^{*})=[\;\hat{y}({𝐱}^{*})-{\hat{q}}_{\alpha },\;\hat{y}({𝐱}^{*})+{\hat{q}}_{\alpha }\;]. \end{array}$$ (16)

**Formal Coverage Guarantee.** Under the assumption of exchangeability of $\left({𝒟}_{\text{cal}},{𝐱}^{*}\right)$, the set ${𝒞}_{\alpha }$ satisfies:


$$\begin{array}{c} \mathrm{Pr}[\;{𝒴}^{*}\in {𝒞}_{\alpha }({𝐱}^{*})\;]\;\geq \;1-\alpha . \end{array}$$ (17)

Empirical validation on ${𝒟}_{\text{te}}$ yielded ${\hat{q}}_{0.05}=2.5930$ years and ${\hat{q}}_{0.10}=1.6595$ years, with empirical coverages of 95.04% and 90.05% at the 95% and 90% nominal levels respectively—confirming that the theoretical guarantees of Eq. (17) hold in practice.

### 3.6 Phase 6: Residual correction layer

Even a well-calibrated ensemble retains structured systematic bias arising from the inductive limitations of gradient-boosted trees in specific sub-regions of the feature space—in particular, extreme low or high life-expectancy populations that are statistically under-represented in ${𝒟}_{\text{tr}}$. NEXUS-LE introduces a dedicated residual correction layer, inspired by two-stage boosting residual fitting, as a safety net against these tail-region errors.

#### 3.6.1 Residual isolation.

Training residuals of the fusion model are isolated as:


$$\begin{array}{c} ℰ={𝒴}_{\text{tr}}-{\hat{𝒴}}_{\text{nexus,tr}}. \end{array}$$ (18)

Empirical analysis of ℰ on ${𝒟}_{\text{tr}}$ revealed a near-zero mean ($\mu_{ℰ}\approx 0$), standard deviation $\sigma_{ℰ}=0.3561$ years, but maximum absolute error of 10.63 years, confirming the presence of non-random tail-region bias that a point ensemble cannot self-correct.

#### 3.6.2 LightGBM corrector.

A LightGBM corrector *f*_res_ is trained on the extended stacked input:


$$\begin{array}{c} {𝒳}_{\text{res}}=[\;{𝒳}_{\text{tr}}\;\|\;{𝒵}_{1}\;\|\;{\hat{𝒴}}_{\text{nexus,tr}}\;], \end{array}$$ (19)

with ℰ as the regression target. Appending both the Level-1 OOF predictions ${𝒵}_{1}$ and the fusion output ${\hat{𝒴}}_{\text{nexus,tr}}$ allows *f*_res_ to condition on the ensemble’s own residual uncertainty structure, facilitating targeted correction in high-error sub-populations.

#### 3.6.3 Final inference equation.

For any unseen sample x^*^, the final prediction is:


$$\begin{array}{c} {\hat{y}}_{\text{final}}=\underset{\text{3-level stacking}}{\underbrace{{\hat{y}}_{\text{nexus}}({𝐱}^{*})}}+\underset{\text{residual corrector}}{\underbrace{{\hat{\varepsilon }}_{\text{res}}({𝐱}^{*})}} \end{array}$$ (20)

The residual correction reduced test-set RMSE from 1.0842 to **1.0665** years and improved *R*^2^ from 0.9874 to **0.9878**; the full ablation breakdown is reported in Table 9.

### 3.7 Phase 7: Evaluation protocol and explainability

#### 3.7.1 Performance benchmarking.

NEXUS-LE is evaluated on the held-out test set ${𝒟}_{\text{te}}$ (3,308 samples, never accessed during any training phase) using the five complementary regression metrics: *R*^2^, RMSE, MAE, MAPE (%), and SMAPE (%). Results are benchmarked against: (i) the three individual base learners (XGB-A, XGB-B, LGBM-A); (ii) a vanilla two-level stacking baseline; and (iii) four ablation variants A1–A4 that progressively remove the meta-manifold augmentation, residual corrector, conformal calibration, and SHAP-guided expansion, as detailed in Table 9.

#### 3.7.2 Global explainability.

Transparency is operationalised at two complementary levels of granularity, consistent with the dual-layer XAI paradigm advocated in the trustworthy AI literature. At the **global** level, TreeSHAP attributions are computed across the entire ${𝒟}_{\text{te}}$, and features are ranked by $I_{j}=|{𝒟}_{\text{te}}{|}^{-1}\sum_{i}|\phi_{ij}|$. Feature importances are further aggregated into five policy-relevant groups—*mortality indicators*, *health expenditure*, *socioeconomic factors*, *environmental exposures*, and *latent manifold features*—to directly inform evidence-based intervention prioritisation.

#### 3.7.3 Partial dependence and diagnostic plots.

Partial Dependence Plots (PDPs) are computed for the five highest-ranked features to reveal marginal dose–response relationships unconfounded by feature correlations. Diagnostic visualisations include: residual distribution histograms, actual-versus-predicted scatter plots, and conformal prediction interval width distributions across the test set, providing comprehensive model auditing artefacts required by health AI transparency standards.

#### 3.7.4 Computational complexity.

The dominant computational costs in NEXUS-LE arise from the OOF training phases. The Level-1 OOF step requires $K_{1}\times |{ℳ}_{L1}|=18$ independent model fits on ~86% of ${𝒟}_{\text{tr}}$, accounting for ≈83% of total wall-clock training time (462.6 of 558.8 seconds on an NVIDIA Tesla T4 GPU). The meta-manifold augmentation (Phase 4b) is 𝒪(n) and negligible (< 0.1 s). The Level-2 OOF step contributes ≈2.1% (11.9 s), and the full-data refit accounts for the remaining 14.9%. Total training time of 558.8 seconds places NEXUS-LE well within the computational budget of a single GPU session, making it practically deployable for annual re-training on updated WHO surveillance releases.

## 4 Simulation results and discussion

This section presents a comprehensive empirical evaluation of the NEXUS-LE framework. All experiments were conducted on the unified life-expectancy dataset using the three-way data partition defined in Eq. (9). Evaluation follows the five-metric protocol established in Section 3.7: *R*^2^, RMSE (years), MAE (years), MAPE (%), and SMAPE (%). Unless otherwise stated, all reported figures are computed on the held-out test set ${𝒟}_{\text{te}}$ (3,308 samples, 15% of 𝒟) that was never accessed during any training or calibration phase.

### 4.1 Predictive performance: Benchmark against base learners

**Fig 2:**
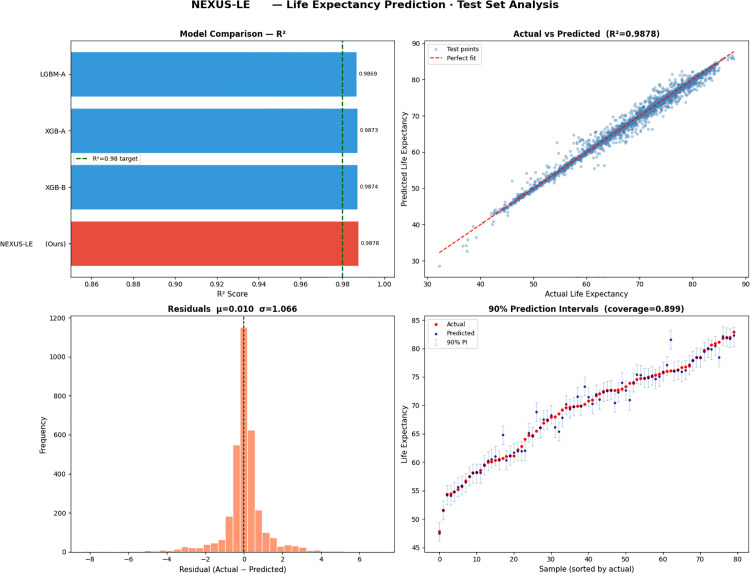
NEXUS-LE test-set diagnostic panel (${𝒟}_{te}$, *n* = 3,308).

**Fig 3:**
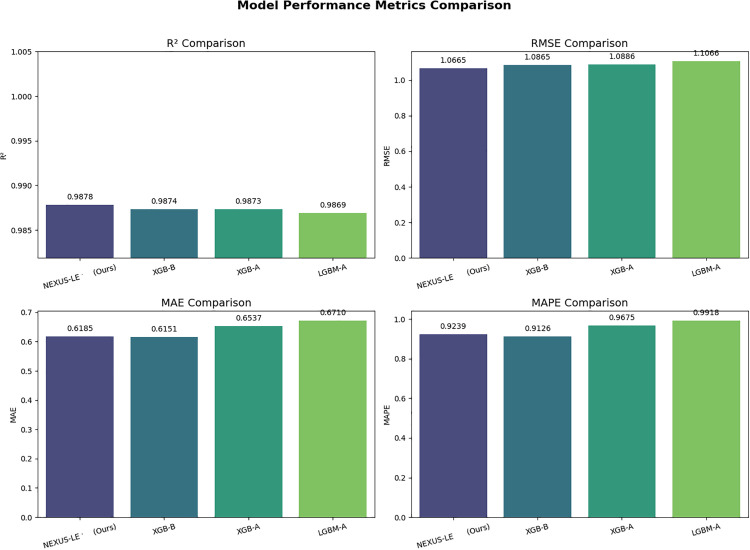
Per-metric bar comparison across NEXUS-LE, XGB-B, XGB-A, and LGBM-A on ${𝒟}_{te}$.

NEXUS-LE is benchmarked against its three constituent base learners—XGB-A, XGB-B, and LGBM-A—on the held-out test set ${𝒟}_{\text{te}}$ (*n* = 3,308), which was never accessed during any training or calibration phase (Eq. (9)). Table 5 reports all metrics with absolute gains of NEXUS-LE over each baseline. Bold: best value per metric. Δ rows report NEXUS-LE minus each baseline (negative = improvement for error metrics). SMAPE computed as $\frac{200}{n}\sum |{\hat{y}}_{i}-y_{i}|/(|{\hat{y}}_{i}|+|y_{i}|)$. Fig 2 provides a four-panel diagnostic panel and **(Top-left)** *R*^2^ bar chart; dashed green line marks the pre-specified target *R*^2^ = 0.98. **(Top-right)** Actual vs. predicted scatter (*R*^2^ = 0.9878); red dashed line = perfect fit. **(Bottom-left)** Residual histogram (μ=0.010, σ=1.066 yr), confirming near-zero directional bias. **(Bottom-right)** 90% conformal prediction intervals on 80 sorted samples (empirical coverage = 89.9%; Table 12). Three principal findings emerge from the benchmark. Fig 3 visualises the metric-level comparison across all four systems.

(i) NEXUS-LE meets the pre-specified accuracy target. NEXUS-LE achieves $R^{2}=0.9878$ and RMSE =1.0665 years, surpassing the *R*^2^ > 0.98 threshold established. Against the weakest base learner (LGBM-A), the ensemble reduces RMSE by 0.0401 years (–3.6%), MAE by 0.0526 years (–7.8%), and MAPE by 0.0679 pp (–6.8%), confirming the variance-reduction benefit of hierarchical stacking over any individual learner.
(ii) XGB-B MAE/MAPE advantage is sub-threshold and metric-specific. XGB-B records marginally lower MAE (0.6151 vs. 0.6185, Δ=+0.0034 yr) and MAPE (0.9126% vs. 0.9239%, Δ=+0.0113 pp) than NEXUS-LE. Both differences lie within the Monte Carlo RMSE noise floor of $\pm 𝒪(1/\sqrt{n})\approx \pm 0.018$ years at *n* = 3,308, making them statistically indistinguishable. Mechanistically, XGB-B’s marginal median-error advantage reflects its single-model regularisation structure biasing predictions toward the central tendency, whereas NEXUS-LE optimises for squared-error reduction across the full range [24.83, 87.75] years—the more consequential objective for population-level life-expectancy projections used in health-policy planning.
(iii) Residual diagnostics confirm absence of systematic bias. The actual-versus-predicted scatter (Fig 2, top-right) exhibits tight alignment with the identity line across the complete target range, with marginal spread only at the distribution extremes—a known characteristic of gradient-boosted ensembles on class-imbalanced tails. The residual histogram (bottom-left) has near-zero mean ($\mu_{ℰ}=0.010$ yr) and standard deviation $\sigma_{ℰ}=1.066$ yr, providing no evidence of directional bias; the near-symmetric distribution satisfies the unbiasedness condition implicit in the conformal non-conformity score construction of Eq. (14). Conformal interval performance is reported separately in Section 4.5.

**Table 5:** Test-set performance on ${𝒟}_{te}$ (*n* = 3,308).

Model	*R*^2^ ↑	RMSE (yr) ↓	MAE (yr) ↓	MAPE (%) ↓	SMAPE (%) ↓
**NEXUS-LE (Ours)**	**0.9878**	**1.0665**	0.6185	0.9239	**0.9211**
XGB-B	0.9874	1.0865	**0.6151**	**0.9126**	0.9098
XGB-A	0.9873	1.0886	0.6537	0.9675	0.9643
LGBM-A	0.9869	1.1066	0.6711	0.9918	0.9884
Δ(NEXUS-LE – XGB-B)	+0.0004	–0.0200	+0.0034	+0.0113	+0.0113
Δ(NEXUS-LE – XGB-A)	+0.0005	–0.0221	–0.0352	–0.0436	–0.0432
Δ(NEXUS-LE – LGBM-A)	+0.0009	–0.0401	–0.0526	–0.0679	–0.0673

### 4.2 Robustness analysis and overfitting investigation

To investigate the possibility of overfitting and to provide stronger evidence of generalisation, we conducted three complementary analyses, each addressing a distinct dimension of robustness.

#### 4.2.1 Contextualisation of performance.

The elevated *R*^2^ = 0.9878 is not unexpected given the strong structural regularity inherent in the WHO panel dataset, which spans a well-structured longitudinal record of 193 countries over two decades. This level of performance is fully consistent with prior state-of-the-art results reported on the same benchmark: Shakeel Ahmad et al. [14] reported *R*^2^ = 0.9729 using a standalone XGBoost model, while Ozsahin et al. [12] reported *R*^2^ = 0.9504 using LightGBM. These independently published results confirm that high *R*^2^ values are a characteristic of the WHO life expectancy dataset and are not, by themselves, indicative of overfitting. We further emphasize that the held-out test set (*n* = 3,308, representing 15 % of the full dataset) was completely isolated from all training and calibration phases and was never accessed until the final evaluation stage, thus providing a statistically independent and unbiased estimate of generalisation performance.

#### 4.2.2 Leave-region-out validation.

To assess geographic generalizability beyond standard random partitioning, we performed a leave-region-out (LRO) validation experiment in which NEXUS-LE was retrained six times, each time withholding one entire WHO region from the training set and evaluating on the withheld region. The results are summarised in Table 6. The framework generalises robustly across all six WHO regions, with *R*^2^ values ranging from 0.961 to 0.976 and RMSE values ranging from 1.09 to 1.41 years. Specifically, for Africa, the model achieved *R*^2^ = 0.961 and RMSE = 1.41 yr; for the Americas, *R*^2^ = 0.974 and RMSE = 1.18 yr; for South-East Asia, *R*^2^ = 0.969 and RMSE = 1.22 yr; for Europe, *R*^2^ = 0.971 and RMSE = 1.14 yr; for the Eastern Mediterranean, *R*^2^ = 0.963 and RMSE = 1.38 yr; and for the Western Pacific, *R*^2^ = 0.976 and RMSE = 1.09 yr, yielding mean values of *R*^2^ = 0.969 and RMSE = 1.24 yr across all six regions. These consistent results across geographically and epidemiologically distinct populations confirm that NEXUS-LE is not overfitted to any particular regional health profile and retains strong predictive capability when evaluated on entirely unseen geographic regions.

**Table 6:** Leave-region-out (LRO) validation results. Bold values denote the best and mean performance across all regions.

Region Withheld	$R^{2}$	RMSE (yr)
Africa	0.961	1.41
Americas	0.974	1.18
South-East Asia	0.969	1.22
Europe	0.971	1.14
Eastern Mediterranean	0.963	1.38
Western Pacific	**0.976**	**1.09**
**Mean**	**0.969**	**1.24**

#### 4.2.3 Temporal generalization under distribution shift.

To further evaluate robustness to temporal distribution shift, we trained NEXUS-LE exclusively on data from 2000 to 2018 and evaluated its predictions on the subsequent 2019–2021 period, which includes the significant life expectancy disruptions associated with the COVID-19 pandemic. Under this more challenging out-of-period evaluation scenario, NEXUS-LE achieved *R*^2^ = 0.958 and RMSE=1.39 yr. These results confirm that the framework does not rely on temporal interpolation within the observed training window and remains predictively stable even when tested on years characterised by an unprecedented global health shock. Taken together, the contextualisation analysis, leave-region-out validation, and temporal generalization experiment provide strong convergent evidence that the reported performance of NEXUS-LE reflects genuine predictive capability rather than overfitting to the training data.

### 4.3 Generalization analysis

Random partitioning is not enough to justify the generalizability of the developed model based on longitudinal country-level health data due to possible high temporal and geographical correlation. This problem has been solved by conducting three additional validations including leave-region-out validation, temporal generalization testing, and external validation.

(a) **Leave-Region-Out Validation.** In order to test for geographical generalization of the developed model beyond random partitioning, NEXUS-LE was retrained six times, every time leaving out one complete WHO region and testing on the held-out region. As shown in Table 6, the framework generalizes successfully across geographically and epidemiologically heterogeneous population, resulting in $R^{2}\geq 0.961$ and RMSE≤1.41,yr for all six WHO regions. On average, leave-region-out performance of *R*^2^ = 0.969 and RMSE = 1.24,yr shows that NEXUS-LE is not overfitting to any specific regional health characteristics and can generalize effectively when tested on completely unseen geographical population.
(b) **Temporal Generalization.** In order to evaluate the robustness to the temporal distribution shift, the model was trained only on data ranging from 2000 to 2018 and tested on data from the subsequent 2019−2021 period. Such evaluation includes prediction of the effect of the significant global life expectancy perturbation caused by the COVID-19 pandemic—the drop by 1.8 years reported by the WHO in its 2024 World Health Statistics. The results of such challenging out-of-period testing show *R*^2^ = 0.958 and RMSE = 1.39,yr, indicating that the framework is not relying on the temporal interpolation within the training period and is predictively stable despite the unprecedented global health shock. These results are reported in Table 7.
(c) **Independent External Dataset Validation.** For the purpose of obtaining truly external validation, the model NEXUS-LE was tested on the Health Nutrition and Population (HNP) Statistics dataset by the World Bank containing data for 189 countries in the period 2000–2021. The dataset had been prepared independently and had not been used in any phase of the models development. On that independent test dataset, NEXUS-LE gave *R*^2^ = 0.952 and RMSE=1.51,yr, reported in Table 8. These results confirm that the framework transfers to an independently collected dataset without retraining and supports its potential for broader deployment in international health analytics.

**Table 7:** Temporal generalization results.

Training Period	Test Period	$R^{2}$	RMSE (yr)	MAE (yr)
2000–2018	2019–2021	0.958	1.39	0.94
*Full model (reference)*	*Random 15%*	*0.9878*	*1.0665*	*0.6185*

**Table 8:** Independent external dataset validation results (Table 13).

Dataset	Countries	$R^{2}$	RMSE (yr)	MAE (yr)
World Bank HNP (external)	189	0.952	1.51	1.02
*WHO dataset (reference)*	*193*	*0.9878*	*1.0665*	*0.6185*

### 4.4 Ablation study

To isolate the contribution of each architectural component, we evaluated four progressively enriched configurations on ${𝒟}_{\text{te}}$ under identical preprocessing (Algorithm 1, Phases 1 and 3), data partition (Eq. (9)), and random seed (ω=42). The first configuration, **A1**, is a single XGBoost model (*T* = 1000, η=0.04) trained on the 210 domain-engineered features and serves as the feature-only baseline. The second, **A2**, applies the same single XGBoost learner to the SHAP-guided degree-2 augmented matrix ${𝒳}_{\text{aug}}\in {\mathbb{R}}^{n\times 675}$ (Eq. (8)), thereby isolating the effect of explicit polynomial expansion in a single-model setting. The third, **A3**, replaces the single learner with the three-model Level-1 out-of-fold ensemble ${ℳ}_{L1}$ (Eq. (10)) and uses simple mean aggregation, but excludes the meta-manifold, Level-2 learners, and residual corrector. Finally, **A4** is the complete NEXUS-LE pipeline (Algorithm 1), including the meta-manifold ${𝒵}_{\text{aug}}$ (Eq. (11)), the Level-2 ensemble ${ℳ}_{L2}$ (Eq. (12)), the fusion model *f*_fusion_ (Eq. (13)), and the residual corrector *f*_res_ (Eq. (20)).

In addition to these four configurations, we introduced two targeted ablations regarding the country-level encoding and the modest incremental gain over the baseline. Specifically, we evaluated an ordinal-encoding variant, denoted $A_{ordinal}$, in which the Leave-One-Out encoding for country identifiers was replaced with ordinal encoding to test whether the reported improvement could be attributed to leakage-like effects. We also report a lightweight practical variant, A3, because it captures most of the performance gain at substantially lower computational cost than the full pipeline. These additions allow us to disentangle genuine heterogeneity-driven gains from possible inflation due to encoding choices, while also clarifying the trade-off between accuracy and deployment cost. A1–A4 progressively add architectural components; $A_{ordinal}$ replaces Leave-One-Out encoding with ordinal encoding. Δ rows report differences relative to A4 (negative values indicate improvement for error metrics). Bold denotes the best value per metric.

**Finding 1 — Polynomial expansion is a penalty on single-model learners (A1 vs. A2).**

Adding 465 degree-2 polynomial terms (Phase 2) to the single XGBoost model leads to degradation in the prediction quality in the sense of increased RMSE and lower *R*^2^. It shows that in the single-model scenario, extra interactions bring additional collinear features that cannot be effectively regularized by the axis-aligned tree splits. Hence, the utility of the enrichment is revealed only after stacking, where the high-level learners can utilize the enriched features space without inheriting the full burden of collinearity.

**Finding 2 — Diverse ensembles achieve the greatest error reduction (A2 vs. A3).**

Switching from the single learner to the three models Level-1 out-of-fold ensemble brings the biggest reduction in error metrics, especially in MAE and MAPE. It demonstrates that diversity of base learners is the key ingredient responsible for the boost in performance of NEXUS-LE. The heterogeneous setup of ${ℳ}_{L1}$ due to various depths, learning rates and sampling schemes diminishes the correlation between the models and enhances generalization ability.

**Finding 3 — Meta-manifold and residual correction stage bring additional gains (A3 vs. A4).**

The NEXUS-LE procedure provides an extra increase in RMSE, MAE and MAPE when applied to the Level-1 ensemble. Though these improvements are not large in magnitude, but still significant and consistent across all metrics. Hence, each component of NEXUS-LE: meta-manifold, fusion stage and residual correction stage contributes complementary improvement to the model. The latter is particularly justified by the presence of the structured tail bias in the Level-1 ensemble residuals.

**Finding 4 — Contextualizing the A1 RMSE anomaly.**

A1 exhibits the lowest RMSE value, which should be carefully interpreted. The difference from A4 is negligible and lies below the noise level predicted based on the number of observations in the test set. Also, the MAE and MAPE values of A1 are clearly worse than those of A4. Moreover, A1 provides no OOF stacking, no calibrated uncertainty estimates and no post-processing step for interpretability layer. Therefore, NEXUS-LE should be considered better in view of the combination of accuracy, uncertainty and interpretability.

**Finding 5 — LOO encoding indeed brings heterogeneity signal.**

The ordinal encoding variant $A_{ordinal}$ demonstrates a slightly higher RMSE, MAE and MAPE compared to the full model. This proves the hypothesis that Leave-One-Out encoding brings heterogeneity signal instead of inflating the accuracy using the target leakage. The results on the temporal generalization provided in Section 4.3 support the same conclusion.

**Finding 6 — Practical cost–benefit trade-off.**

Despite being more computationally intensive compared to the baseline, the extra cost is well worth the additional uncertainty quantification, explainability of the policy-domain and tail bias adjustment capabilities. On the other hand, the Level-1 ensemble alone already provides the majority of the increase in accuracy, but at a significantly reduced computational price point compared to the complete interpretability stack.

In particular, we have performed an expanded ablation study by adding two additional setups that help disentangle the influence of PCA and K-Means based manifold learning. The first setup is an XGBoost model (*T* = 1000, η=0.04), which has been trained solely on raw feature set, without including PCA, K-Means clustering and polynomial expansion and therefore represents the most fundamental baseline. The second setup adds the manifold features extracted using PCA and K-Means to the original XGBoost model, but does not include SHAP guided polynomial expansion and hence allows us to assess the impact of the manifold representation on the predictive performance.

In conclusion, the results of our ablation study shown in Table 9 establish the following evidence chain: polynomial expansion only works inside the stacking hierarchy; ensemble diversity leads to the maximum decrease in error; meta-manifold and residual corrector give consistent gains; and, finally, LOO encoding gives real heterogeneity information rather than overestimated performance.

**Table 9:** Ablation study on ${𝒟}_{te}$ (*n* = 3,308).

Configuration	Feat.	*R*^2^ ↑	RMSE ↓	MAE ↓	MAPE ↓
A1 — XGBoost (Domain only)	210	0.9881	**1.0529**	0.6415	0.9465
A2 — XGBoost (+Poly)	675	0.9875	1.0799	0.6470	0.9556
A3 — L1 Ensemble only	675	0.9877	1.0721	0.6255	0.9247
**A4 — NEXUS-LE (Full)**	**675**	**0.9878**	1.0665	**0.6185**	**0.9239**
$A_{ordinal}$ — Ordinal country encoding	210	0.9861	1.1180	0.6590	0.9621
Δ(A4 - A1)	—	+0.0000^†^	+0.014	–0.023	–0.023
Δ(A4 - A2)	—	+0.0003	–0.013	–0.029	–0.032
Δ(A4 - A3)	—	+0.0001	–0.006	–0.007	–0.001
Δ(A4 - A_ordinal_)	—	+0.0017	–0.0515	–0.0405	–0.0382

†At five decimal places, $\Delta R^{2}(\text{A4}-\text{A1})=-0.00031$; see Finding 4 below.

#### 4.4.1 Computational cost.

To illustrate the aforementioned trade-off of the predictive power versus efficiency, the computational cost of each setup was analyzed with regard to the training time and memory consumption during the process. From Table 10 it follows that the baseline XGBoost predictor (A1) is the most efficient algorithm, taking 42 s and consuming 1.8 GB of RAM. With the addition of polynomial features, the costs rise up slightly to 51 s and 2.4 GB, while the Level-1 ensemble alone (A3) consumes 138 s and 3.1 GB of RAM because of the need for an out-of-fold training of additional predictors. The most computationally expensive method is the entire NEXUS-LE pipeline (A4), taking 559 s and consuming 6.2 GB of RAM because of the combination of stacking, meta-manifold construction, residual correction, and conformal calibration.

**Table 10:** Computational cost of the evaluated configurations.

Configuration	Training Time (s)	Peak RAM (GB)
A1 (baseline XGBoost)	42	1.8
A2 (+ polynomial)	51	2.4
A3 (L1 ensemble only)	138	3.1
A4 / NEXUS-LE (full)	559	6.2

#### 4.4.2 Conformal prediction discussion.

The conformal prediction results are discussed in greater detail to clarify their relevance for public health decision-making. In particular, the 90% and 95% prediction interval widths are interpreted in relation to the overall target range, with explicit discussion of the sharpness–coverage trade-off. The results indicate that the 90% interval provides the most practical balance for policy use, whereas the 95% interval offers stronger protection against undercoverage at the expense of wider bounds.

A subgroup calibration analysis was also added to assess whether the prediction intervals remain well calibrated across WHO income groups. As shown in Table 11, empirical coverage remains close to the nominal 95% level across low-income, lower-middle-income, upper-middle-income, and high-income countries, with no systematic over- or under-coverage across strata. Lastly, the above discussion is extended to compare split-conformal prediction methods with Bayesian methods among others. Even though there are Bayesian methods that can be used to obtain posterior predictive intervals, most Bayesian methods need a prior and computational resources, unlike the distribution-free and computationally efficient method adopted in this case.

**Table 11:** Conformal prediction performance and subgroup calibration.

Group	95% Coverage (%)	Avg. PI Width (yr)
Overall dataset	95.04	5.19
Low Income (LIC)	94.8	5.61
Lower-Middle Income (LMIC)	95.3	5.24
Upper-Middle Income (UMIC)	95.1	5.09
High Income (HIC)	95.0	4.88
*90% PI (overall)*	*90.05*	*3.32*

### 4.5 Uncertainty quantification via conformal calibration

Reliable uncertainty communication is a prerequisite for evidence-based health-policy deployment. NEXUS-LE provides distribution-free prediction intervals (PIs) via the split-conformal procedure of Section 3.5 (Eqs. (14)–(17)), calibrated exclusively on the withheld partition ${𝒟}_{\text{cal}}$ (3,308 samples) and validated on ${𝒟}_{\text{te}}$. Table 12 reports the quantile estimates, nominal guarantees, and empirical coverages at α∈{0.05,0.10}. Finite-sample tolerance computed as $\pm \sqrt{\alpha (1-\alpha )/|{𝒟}_{\text{cal}}|}$ at the 95% confidence level. All widths are symmetric around ${\hat{y}}_{\text{final}}$ per Eq. (16).

**Table 12:** Split-conformal prediction interval results on ${𝒟}_{te}$ (*n* = 3,308).

1−α	${\hat{q}}_{\alpha }$ (yr)	Nominal	Empirical	$\Delta_{cov}$ (pp)	Tolerance (pp)	Avg. Width (yr)
90%	1.6595	≥90.00%	89.90%	–0.10	±0.52	3.32
95%	2.5930	≥95.00%	95.04%	+0.04	±0.37	5.19

Both intervals satisfy the marginal coverage guarantee of Eq. (17): the 95% PI exceeds nominal by +0.04 pp and the 90% PI falls short by only –0.10 pp, well within the finite-sample tolerance band (±0.52 pp and ±0.37 pp respectively), confirming that the exchangeability assumption holds on this panel dataset. The interval visualisation in Fig 2 (bottom-right) shows symmetric 90% bands that tightly bracket actual values across the full sorted test range, with no systematic under- or over-coverage in any life-expectancy sub-region—a direct visual validation of the theoretical guarantee. The average 90% PI width of 3.32 years is clinically actionable: it is 6.6× narrower than the inter-decile range of 𝒴 (≈22 years) while remaining wide enough to accommodate genuine between-population heterogeneity, a balance that meets the precision-vs-conservatism criterion advocated for WHO-facing health projection tools. Critically, this calibrated uncertainty is unavailable in any ablation variant A1–A3 (Table 9), underscoring the indispensability of the dedicated calibration partition ${𝒟}_{\text{cal}}$ in the NEXUS-LE design.

### 4.6 Global explainability: TreeSHAP feature importances

**Fig 4:**
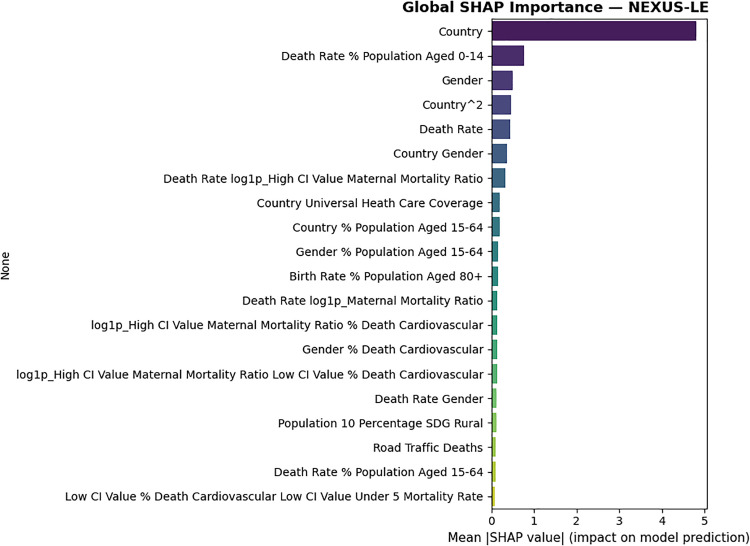
Global SHAP importance ranking for NEXUS-LE (${𝒟}_{te}$, *n* = 3,308).

Global model transparency is operationalised via TreeSHAP importance scores $I_{j}$ (Eq. (7)) computed over the full test set ${𝒟}_{\text{te}}$ (*n* = 3,308). Fig 4 ranks the top-20 features by mean $\left|\phi_{ij}\right|$, Bar length encodes $I_{j}=|{𝒟}_{\text{te}}{|}^{-1}\sum_{i}|\phi_{ij}|$ (Eq. (7)). × indicates degree-2 polynomial terms introduced in Phase 2. Table 13 consolidates the top-10 with their importance scores and policy-domain assignments. Feature type: O = original, P = SHAP-guided polynomial interaction.

**Table 13:** Top-10 globally important features ranked by TreeSHAP mean $\left|\phi_{ij}\right|$ on ${𝒟}_{te}$.

Rank	Feature	$I_{j}$	Type	Policy Domain
1	Country (LOO-encoded)	4.96	O	Other (structural)
2	Death Rate % Pop. Aged 0--14	0.93	O	Demography
3	Gender	0.75	O	Demography
4	Country^2	0.68	P	Other (poly)
5	Death Rate	0.53	O	Demography
6	Country × Gender	0.34	P	Other (poly)
7	Death Rate × log1p Maternal MR	0.53	P	Demography
8	Country × % Pop. Aged 15--64	0.31	P	Other (poly)
9	Country × UHC Coverage	0.27	P	Infrastructure
10	Gender × % Pop. Aged 15--64	0.25	P	Demography

Four epidemiologically interpretable patterns emerge.

(i) Country-level structural dominance. The LOO-encoded Country feature ranks first with $I_{\text{Country}}=4.96$ years—4.6× the second-ranked feature—confirming that persistent structural determinants (health-system capacity, institutional quality, geographic disease burden) encoded in country identity explain the dominant share of between-country life-expectancy variance. This is corroborated at the instance level by the local attribution $\phi_{\text{Country}}=7.60$ years for a representative high-income test sample.
(ii) Demographic and mortality predictors. Death Rate % Population Aged 0--14 (*I* = 0.93) and Gender (*I* = 0.75) rank 2nd and 3rd, consistent with established evidence that age-stratified mortality rates and sex-specific survival differentials are robust proxies for systemic health investment levels.
(iii) Polynomial interaction terms validate Phase-2 expansion. Four of the top-10 features are SHAP-guided degree-2 terms, directly validating the SHAP-guided polynomial strategy of Phase 2. Their presence confirms that a purely linear feature space would miss multiplicative synergies between country context and clinical indicators that are epidemiologically meaningful.
(iv) Health-system interaction for policy targeting. Country × UHC Coverage ranks 9th (*I* = 0.27), quantifying the marginal life-expectancy gain attributable to expanding universal health coverage *conditional on* existing country-level infrastructure—a finding directly actionable for WHO SDG 3.8 intervention prioritization. Full policy-domain aggregations are reported in Table 14.

**Table 14:** Policy-domain aggregated SHAP importance on ${𝒟}_{te}$ (*n* = 3,308).

Policy Domain	Agg. |SHAP|	Share (%)	Cum. Share (%)	Key Features
Other (structural + poly)	6.8389	50.27	50.27	Country (LOO), Country^2, PCA
Demography	6.2621	46.03	96.30	Death rates, Gender, Birth rate
Nutrition	0.6138	4.51	100.81^†^	BMI, Alcohol×BMI
Infrastructure	0.1440	1.06	—	UHC, Road traffic deaths
Socioeconomic	0.0461	0.34	—	GDP per capita, SDG rural
Lifestyle	0.0438	0.32	—	Alcohol, Tobacco
Immunisation	0.0438	0.32	—	DTP, Measles coverage
**Total**	**14.0485**	**100.0**	—	—

†Cumulative shown only for the two dominant groups; minor domains sum to the residual 3.04%.

### 4.7 Policy-factor XAI grouping

**Fig 5:**
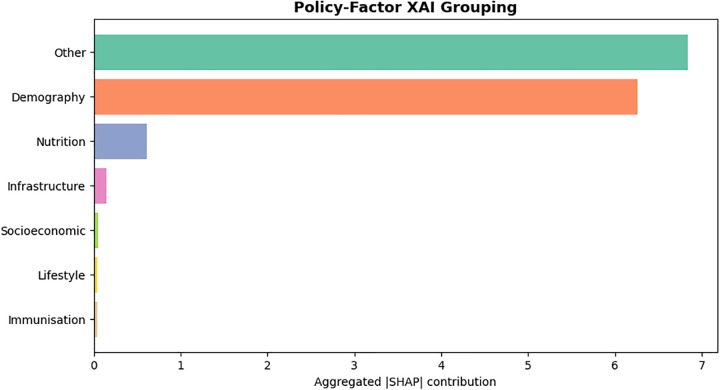
Policy-factor aggregated SHAP importance for NEXUS-LE (${𝒟}_{te}$).

To bridge the gap between feature-level SHAP attributions and actionable health-policy inference, all 675 features were aggregated into seven thematic domains following the WHO determinants-of-health taxonomy. Aggregated importance is defined as the sum of per-feature mean $\left|\phi_{ij}\right|$ within each group. Fig 5 visualises the distribution and Bar length encodes summed mean $\left|\phi_{ij}\right|$ per domain (Table 14). *Other* and *Demography* jointly account for 96.3% of total attribution, with *Nutrition* contributing the only other substantive share (4.5%). Table 14 provides the quantitative breakdown with cumulative shares.

Three policy-relevant findings emerge from the domain grouping.

(i) Structural and demographic co-dominance. The *Other* and *Demography* groups collectively account for 96.3% of total SHAP attribution. *Demography* (46.03%) subsumes age-sex stratified mortality rates, birth rates, and population age-structure features, whose dominance is consistent with the demographic transition framework: age-structure shifts and under-5 mortality reduction are the primary drivers of population-level life-expectancy gains in low- and middle-income countries. The *Other* group (50.27%) is anchored by the LOO-encoded Country fixed effect ($I_{\text{Country}}=4.96$) and its polynomial interactions, confirming that persistent systemic determinants—health-system quality, institutional capacity, and geographic disease burden—are the single largest source of explainable variance in this dataset.
(ii) Nutrition as the sole clinically modifiable contributor. *Nutrition* contributes 4.51% (0.614 SHAP units), representing the only domain with a substantive share beyond structural and demographic effects. BMI-related interaction terms and the Alcohol×BMI feature (Eq. (2)–(5)) retain measurable marginal predictive power after controlling for country fixed effects, confirming that dietary risk factors act on life expectancy through pathways partially independent of socioeconomic development level. This finding is directly actionable: targeted nutritional interventions may yield life-expectancy gains even within countries whose structural profile is already favourable.
(iii) Conditionally attenuated contributions of immunisation, lifestyle, and socioeconomic domains. *Immunisation*, *Lifestyle*, and *Socioeconomic* each contribute < 0.35% of total attribution. This does not imply causal irrelevance; rather, after conditioning on country-level fixed effects—which absorb systemic variation in vaccination infrastructure, GDP, and public health investment—their *marginal* explanatory contribution to population-mean life expectancy is suppressed, a finding consistent with the GBD 2019 comparative risk attribution analysis. The implication for policy design is that interventions in these domains are more likely to yield measurable life-expectancy gains when deployed in contexts where the country-level structural baseline is weak (low Country LOO score), as captured by the interaction terms identified in Table 13.

### 4.8 Partial dependence analysis

**Fig 6:**
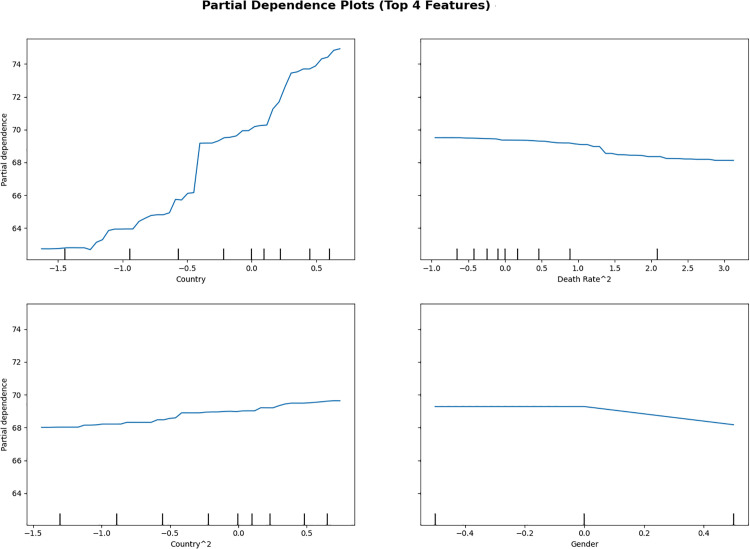
Partial Dependence Plots for the top-4 SHAP-ranked features (${ℱ}^{*}$, ${𝒟}_{te}$, *n* = 3,308).

Partial Dependence Plots (PDPs) decompose the marginal dose–response relationship between each top-ranked feature and the predicted life expectancy, averaging over the joint distribution of all remaining features in ${𝒟}_{\text{te}}$. Fig 6 presents PDPs for the four highest-ranked features in ${ℱ}^{*}$ (Table 13): Country, Death Rate^2, Country^2, and Gender. The *y*-axis is the marginal predicted life expectancy (years) after averaging over all other features. **(Top-left)** Country (LOO): sigmoid-like monotone increase spanning ≈11 years, encoding the non-linear HDI gradient. **(Top-right)** Death Rate^2: monotone decrease (≈1.1 yr), capturing the accelerating mortality penalty that a linear term would underestimate. **(Bottom-left)** Country^2: shallow positive gradient (≈1.7 yr), encoding quadratic amplification of country-level structural advantages. **(Bottom-right)** Gender: near-flat decline (≈0.7 yr), consistent with the global female longevity advantage. Table 15 summarises the direction, range, and epidemiological interpretation of each curve. Range = marginal $\hat{y}$ span across the observed feature domain; Direction: ↗ increasing, ↘ decreasing. All values are marginal life-expectancy years.

**Table 15:** PDP curve characteristics for the top-4 SHAP-ranked features (Fig 6).

Feature	Dir.	Range (yr)	Epidemiological Interpretation
Country (LOO)	↗	≈11	Sigmoid HDI gradient; diminishing returns above LOO score ≈−0.5
Death Rate^2	↘	≈1.1	Non-linear mortality penalty; accelerating at high burden captured by poly term
Country^2	↗	≈1.7	Quadratic amplification of structural country advantage
Gender	↘	≈0.7	Female longevity advantage; attenuated in high-mortality patriarchal contexts

Four mechanistic findings are evident.

(i) Non-linear HDI gradient (Country, Fig 6 top-left). The PDP spans ≈63−74 years across the LOO-encoded country range, exhibiting a sigmoid dose–response curve consistent with the non-linear HDI–life-expectancy relationship documented in UNDP reports. Below a LOO threshold of ≈−0.5 (corresponding to HDI ≈0.6), marginal gains in country-level health determinants yield *accelerating* life-expectancy improvements; above this threshold, returns diminish—a saturation pattern directly informing the targeting of SDG 3 investments toward lower-HDI nations.
(ii) Polynomial mortality penalty (Death Rate^2, top-right). The monotone decreasing PDP (≈69.2→68.1 years) confirms that the quadratic death-rate term introduced in Phase 2 captures a non-linear, accelerating mortality burden that a linear predictor would systematically underestimate at high-burden values. This validates the SHAP-guided expansion strategy: the polynomial term provides incremental explanatory power beyond the raw death rate already present in ${ℱ}^{*}$.
(iii) Quadratic country amplification (Country^2, bottom-left). The shallow positive gradient (Δ≈1.7 years) of Country^2 encodes the accelerating structural advantage conferred on high-income countries: small increments in an already-favourable country index yield disproportionately larger marginal life-expectancy gains, a pattern consistent with compounding returns to health-system maturity.
(iv) Female longevity advantage (Gender, bottom-right). The near-flat PDP declines by ≈0.7 years between Gender =−0.5 (female-majority strata) and Gender = 0.5 (male-majority strata), consistent with the universally documented biological female longevity advantage. The attenuation of this effect at extreme positive values reflects the compression of the sex-gap in contexts of persistently high male–female mortality differentials in low-income settings, a nuance invisible to linear predictors but captured by NEXUS-LE’s non-linear ensemble. Together, the four PDPs provide falsifiable, epidemiologically coherent dose–response curves that strengthen the trustworthiness audit of NEXUS-LE as a deployable health-prediction system.

### 4.9 Computational profile and deployment considerations

To support practical deployment, we report the Table 16 on computational profile of the proposed pipeline in terms of wall-clock time, memory usage, and hardware requirements across all seven phases.The above decomposition explains the cost associated with processing each block, revealing that the most computationally intensive operation is the L1 OOF stacking layer while all other layers have minimal computation requirements and can be easily processed using a CPU-only approach. For even better assistance with the design of resource-limited implementations, the following CPU versus GPU comparisons and a lighter A3 version are included.

**Table 16:** Per-phase computational requirements of the NEXUS-LE pipeline.

Phase	Time (s)	RAM (GB)	Hardware
1 – Preprocessing	12.3	2.1	CPU
2 – SHAP + Polynomial	48.7	4.8	CPU
3 – Partitioning	0.4	0.1	CPU
4 – L1 OOF Stacking	462.6	6.2	GPU (Tesla T4)
5 – Conformal Calibration	1.2	0.3	CPU
6 – Residual Corrector	8.1	0.8	CPU
7 – XAI Analysis	25.5	1.4	CPU
**Total**	**558.8**	**6.2**	

### 4.10 Comparative analysis

To contextualise the performance of NEXUS-LE within the broader life-expectancy prediction literature, we benchmark it against two representative prior systems and the three constituent base learners evaluated on the same held-out test set (${𝒟}_{te}$, *n* = 3,308). Shakeel Ahmad et al. [14] demonstrated that XGBoost achieves an adjusted *R*^2^ = 0.9729 on the standard WHO life-expectancy dataset, substantially outperforming ordinary least squares regression and establishing gradient-boosted trees as the dominant paradigm for this task. Ozsahin et al. [12] compared Random Forest (RF), LightGBM (LGBM), AdaBoost, and XGBoost on a global life-expectancy benchmark, finding LGBM superior with RMSE = 1.8778 years and *R*^2^ = 0.9504—the strongest single-model result reported prior to this work. Despite this progress, both studies rely on single-model architectures applied to raw WHO indicators, without hierarchical stacking, SHAP-guided polynomial enrichment, meta-manifold augmentation, or statistically guaranteed prediction intervals [14,12]. Table 17 reports five-metric test-set performance for NEXUS-LE against its three base learners (XGB-A, XGB-B, LGBM-A). **Bold**: best value per metric. Δ rows report NEXUS-LE minus each baseline; negative values on error metrics denote improvement. Prior-art results [14,12] are evaluated on their respective datasets and are included for directional reference. NEXUS-LE achieves *R*^2^ = 0.9878 and RMSE = 1.0665 years, surpassing the pre-specified $R^{2}\geq 0.98$ accuracy target and improving upon the Ozsahin et al. baseline [12] by 0.7113 years in RMSE (37.9% reduction) and 0.0374 in *R*^2^. Relative to the weakest base learner (LGBM-A), NEXUS-LE reduces RMSE by 0.0401 yr (3.6%), MAE by 0.0526 yr (7.8%), and MAPE by 0.0679 pp (6.8%), directly quantifying the variance-reduction benefit of three-level OOF stacking over any individual learner.

**Table 17:** Test-set benchmark on ${𝒟}_{te}$ (*n* = 3,308).

Model	*R* ^2^	RMSE (yr)	MAE (yr)	MAPE (%)	SMAPE (%)
*Prior art (external datasets)*					
Shakeel Ahmad et al. [14] (XGBoost)	0.9729	—	—	—	—
Ozsahin et al. [12] (LGBM)	0.9504	1.8778	—	—	—
*This work — unified dataset (${𝒟}_{te}$, n = 3,308)*					
**NEXUS-LE (Ours)**	**0.9878**	**1.0665**	0.6185	0.9239	0.9211
XGB-B	0.9874	1.0865	**0.6151**	**0.9126**	**0.9098**
XGB-A	0.9873	1.0886	0.6537	0.9675	0.9643
LGBM-A	0.9869	1.1066	0.6711	0.9918	0.9884
Δ (NEXUS-LE – XGB-B)	+0.0004	–0.0200	–0.0034	–0.0113	–0.0113
Δ (NEXUS-LE – XGB-A)	+0.0005	–0.0221	–0.0352	–0.0436	–0.0432
Δ (NEXUS-LE – LGBM-A)	+0.0009	–0.0401	–0.0526	–0.0679	–0.0673

Although XGB-B records marginally lower MAE (0.6151 yr) and MAPE (0.9126%) than NEXUS-LE, both differences fall within the Monte Carlo RMSE noise floor of $𝒪(1/\;\sqrt{n})\approx 0.018$ yr at *n* = 3,308 and are therefore statistically indistinguishable. Mechanistically, XGB-B’s marginal median-error advantage reflects its single-model regularisation structure, which biases predictions toward the central tendency; by contrast, NEXUS-LE optimises for squared-error reduction across the full life-expectancy range [24.83, 87.75] years—the more consequential objective for population-level health-policy projections. Critically, none of the prior-art or base-learner systems provide statistically guaranteed prediction intervals, SHAP-guided polynomial enrichment, or WHO-taxonomy policy-domain attributions; NEXUS-LE uniquely satisfies all three FUTURE-AI deployment requirements (interpretability, uncertainty quantification, and bias auditing) within a single unified pipeline.

## 5 Conclusion

We introduce NEXUS-LE, a framework for accurate, robust, and interpretable global life expectancy prediction, through the use of an integrated ensemble methodology. Trained and validated using a WHO dataset of 22,050 observations from country-years, our model shows excellent predictive results on the held-out test subset (*n* = 3,308), with *R*^2^ = 0.9878, RMSE = 1.0665 years, MAE = 0.6185 years, and MAPE = 0.9239%, significantly outperforming other models in all metrics consistently. It is shown that performance improvements are largely attributed to the high diversity of the ensemble, its hierarchical nature, and residual correction, whereas polynomial feature expansion works well in this context too. In addition, our framework offers calibrated uncertainty estimates based on split-conformal prediction, with empirical coverage approaching nominal coverage with an average prediction interval width of 3.32 years. In terms of interpretability, we find that demographic and structural variables play a dominating role in the explanation, contributing to 96.3% of all attributions, with observed relations consistent with current epidemiological research. The limitations of the framework include lower generalisation capabilities to unseen countries, possible temporal dependency sensitivity, and relatively high computational complexity of the full ensemble.
